# Evaluation of the hepatoprotective effect of Yigan mingmu oral liquid against acute alcohol-induced liver injury in rats

**DOI:** 10.1186/s12906-020-2817-9

**Published:** 2020-02-05

**Authors:** Qigui Mo, Gao Zhou, Baibo Xie, Bingxin Ma, Xinyu Zang, Yuxin Chen, Linyou Cheng, James Hua Zhou, Youwei Wang

**Affiliations:** 1 Institute of TCM and Natural Products, School of Pharmaceutical Sciences, Wuhan University, Wuhan, 430071 People’s Republic of China; 2 Beijing Hebabiz Biotechnology Co. Ltd, Beijing, 102206 People’s Republic of China; 3 Guangxi Hebabiz Pharmaceutical Co. Ltd, National and Region joint Engineering Center for Anticancer Drug Development, Qinzhou, 535008 People’s Republic of China; 4 MOE Key Laboratory of Combinatorial Biosynthesis and Drug Discovery, Wuhan University, Wuhan, 430072 People’s Republic of China

**Keywords:** Yigan mingmu oral liquid, Hepatoprotective, Alcohol, Alcoholic liver disease, Oxidative stress

## Abstract

**Background:**

Yigan mingmu oral liquid (YGMM) is a herbal medicine based on a famous Chinese herbal formula that has been used for sore eyes for more than 400 years. Eye health is closely associated with the liver based on TCM. This study aimed to investigate the hepatoprotective effect of YGMM against acute liver injury induced by alcohol in rats.

**Methods:**

Experimental rats were administered with silymarin and YGMM through the gastric gavage during the entire experiment. Starting from the 11th day, the rats were administered orally with 14 ml/kg Red Star Erguotou Liquor, a popular brand, at 4 h after the dose of silymarin (100 mg/kg) and YGMM (1, 2.5 and 5 ml/kg in low, middle and high dosage group, respectively) once a day for 4 weeks except for the rats in the normal group. Biochemical parameters, including ALT, AST, TB, TG, T-SOD, GSH, and MDA were detected to evaluate the protective effect of YGMM. Pathological changes were observed through histopathological examination.

**Results:**

Treatment with YGMM exhibited a significant protective effect by reversing the biochemical parameters (ALT, AST, TB, TG, and GSH) and histopathological changes. Histopathological examination by Oil Red O Staining Solution showed that lipid droplets were significantly reduced in the silymarin and YGMM groups (*p* < 0.001) when compared to alcohol group.

**Conclusions:**

YGMM exhibits a significant hepatoprotective activity against acute liver injury induced by alcohol in rats.

## Background

Alcoholic liver disease (ALD) has recently become an important liver disease due to the increasing levels of alcohol consumption in the world [1]. The severity of this disease increases in the time and dose dependent manner with alcohol consumption and ranges initially from steatosis and steatohepatitis to fibrosis and cirrhosis [2–4]. However, the exact pathogenic mechanisms of ALD remain unclear. Previous studies have suggested that mitochondrial damage, generation of free radicals, and oxidative stress are important pathogenic events in the development of ALD [5, 6]. Supportive care and abstinence from alcohol are the only effective treatment methods. Moreover, some synthetic drugs, including bicyclol, tiopronin, and bifendate, have been used to alleviate the symptoms of ALD [7, 8]. Nevertheless, the definite and practical treatment strategies for ALD are still ambiguous. Developing traditional Chinese formulas and natural products with hepatoprotective effects has recently drawn attention [9, 10]. Given their multi-targeted and less toxic features, many herbal medicines have been investigated for ALD treatment [11].

Yigan mingmu oral liquid (YGMM) is a herbal medicine based on a famous Chinese herbal formula composed of 12 crude herbs: Rehmanniae Radix Preparata (Shudihuang), Angelicae Sinensis Radix (Danggui), Lycii Fructus (Gouqizi), Paeoniae Radix Alba (Baishao), Ophiopogonis Radix (Maidong), Chrysanthemi Flos (Juhua), Anemones Altaicae Rhizoma (Jiujiechangpu), Polygonati Odorati Rhizoma (Yuzhu), Chuanxiong Rhizoma (Chuanxiong), Citri Reticulatae Pericarpium (Chenpi), Cassiae Semen (Juemingzi), and Bupleuri Radix (Chaihu). This formula was approved by the CFDA as a therapeutic drug for sore eyes, lumbar debility, dizziness, hypomnesis, and body fatigue.

YGMM is based on *Siwu decoction*, an ancient formula first recorded in the secret formulary for traumatology and fracture taught by immortal in the Tang Dynasty and was used to treat trauma and extravasated blood. *Siwu decoction* was also found in some famous ancient medical books as treatment for obstetrical and gynecological diseases over the last hundred years. These books chronologically included *Prescriptions of Peaceful Benevolent Dispensary* in the Song Dynasty, *Medical Formulae Investigations* by Wu Kun in the Ming Dynasty, and *Detailed Outline for Benefiting Female* by Wu Zhiwang in the Ming Dynasty. *Siwu decoction* was initially composed of four Chinese herbals, namely, Angelicae Sinensis Radix, Chuanxiong Rhizoma, Paeoniae Radix Alba, and Rehmanniae Radix Preparata. In addition to the above herbals, Notopterygii Rhizoma et Radix, Saposhnikoviae Radix, and Angelicae Dahuricae Radix were incorporated in another famous formula named as *Angelicae Sinensis Tonic Decoction*, which was recorded in *Shen Shi Yao Han* by Fu Renyu in the Late Ming Dynasty and is usually used for sore eyes. Through incessant development and optimization, this formula was popularized in the Qing Dynasty and is still useful today. According to TCM, eye health is closely associated with the liver. *Huang Di Nei Jing*, one of the most famous ancient medical books in China, states that liver-qi is connected with the eyes; hence, the eyes can distinguish five colors when the liver-qi is unobstructed.

Pharmacological studies have showed that oxidative stress is an important pathogenesis for ALD [12–15]. Most of herbs in YGMM possess significant antioxidant activity [16–21], and some of them have been used for some diseases because of this property [22, 23]. More specifically, ferulic acid isolated from Angelicae Sinensis Radix and Chuanxiong Rhizoma possesses antioxidant activity and protection against alcohol-induced liver injury [24, 25]. Rehmanniae Radix Preparata, Lycii Fructus, and Paeoniae Radix Alba show antioxidant or anti-inflammatory activity [26–28]. Chrysanthemi Flos and Bupleuri Radix can also prevent liver injury [29, 30]. Besides, fat accumulation mainly results from lipid metabolic disorder, and is commonly observed in human and animal with ALD [31–33]. Bupleuri Radix and Polygonati Odorati Rhizoma could ameliorate fat metabolic disorder [34, 35], *Ophiopogon japonicus* and Paeonia moutan could suppress the hepatic lipid accumulation [36, 37]. Based on the above findings, we speculatedconjecture that YGMM could has hepatoprotective effects on the alcohol-induced liver injury.

In this study, the protective effects of YGMM on acute alcohol-induced liver injury in rats were investigated. The characteristics of liver injury were estimated by AST, ALT, and histopathological changes. In addition, T-SOD, GSH, MDA, TB, and TG levels in the hepatic tissues were detected to identify the possible mechanisms.

## Methods

### YGMM

YGMM samples were provided by Hebabiz Pharmaceutical (Guangxi, China). In brief, six TCM herbs, including Angelicae Sinensis Radix, Chrysanthemi Flos, Anemones Altaicae Rhizoma, Chuanxiong Rhizoma, Citri Reticulatae Pericarpium, and Bupleuri Radix, were crushed, from which the volatile oil was extracted by water distillation, meanwhile the filtrateI and residues were collected for further use. The residues and the rest six TCM herbs were decocted with water, and the decoction was filtered to obtain filtrate II. FiltrateI was combined with filtrateII, then concentrated into an ointment. The ointment was redissolved with ethanol, then the supernatant was concentrated after evaporating ethanol into a recovery tank. The concentrated supernatant was mixed with appropriate amount of water, honey, ethyl paraben and the collected volatile oil to produce YGMM.

### Animals

Male Sprague–Dawley rats (200 ± 20 g) were bought from the Center for Animal Experiment of Wuhan University, Wuhan, China. These experimental rats were allowed to adapt to feeding conditions for 1 week prior to the experiments. The feeding conditions were maintained at 25 ± 2 °C, 40–70% relative humidity, and 12 h dark/light cycle. The rats had free access to standard rodent pellet diet and water during the assay period. The experimental protocol was approved by the Animal Ethics Committee of Wuhan University, Wuhan, China. Animal assays were conducted according to the guidelines of the Committee for the Purpose of Control and Supervision of Experiments on Animals.

### Drug administration

**Fig. 1:**
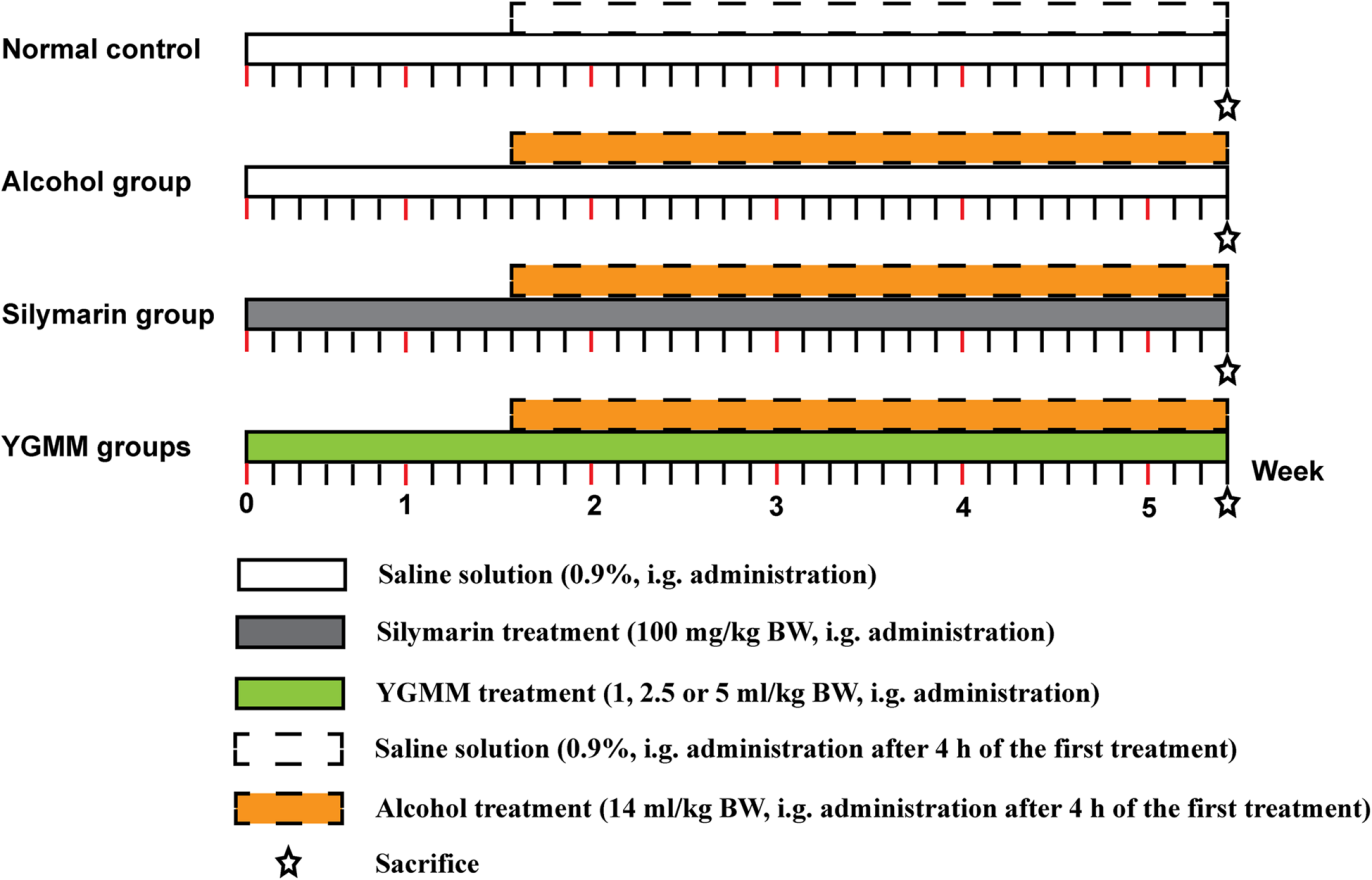
Experimental timelines

After 1 week adaptation, the rats were assigned to the following six groups (six animals each) based on their body weight (BW): normal control, alcohol group, Silymarin group (as the positive control), and YGMM groups. According to preliminary experiments, the following daily doses were adopted for each treated group: the low, medium, and high YGMM doses were 1, 2.5, and 5 ml/kg BW, respectively, whereas silymarin dose was 100 mg/kg BW. The experimental process was showed in Fig. 1. In the beginning, the rats in the YGMM group were given a gavage of YGMM in three different doses, whereas those in the silymarin group were simultaneously fed with silymarin (Madaus GmbH, Cologne, Germany) through gastric gavage. The rats in the normal control and alcohol groups were given equal volumes of physiological saline. At the 11th day, after 4 h of treatment as mentioned above, all groups (except for the control group) were given a gavage of 14 ml/kg BW of Red Star Erguotou Liquor (Beijing Red Star, Beijing, China) once a day for 4 weeks. The liquor contains 56% alcohol, so the dose approximately equals to 7.84 g alcohol per kilogram body weight. After the last treatment (day 38), the rats were fasted for 4 h and were executed by the cervical dislocation method. Blood samples were collected immediately and allowed to clot. The serums were obtained through centrifugation at 3000 rpm for 8 min at 4 °C and were placed in ultra-low-temperature freezer until the assay of AST, ALT, TB, and TG. The liver and kidney were excised, cleared of blood with ice-cold saline, and stored in a refrigerator immediately until the assay of T-SOD, GSH, and MDA.

### Biochemical assay

AST, ALT activities and TB (Nanjing Jiancheng, Nanjing, China), TG (Shanghai Mind, Shanghai, China) levels were detected using the assay kits guided by the corresponding protocols.

Liver and kidney homogenates (10%, w/v) were prepared by using a high-speed dispersator (Ningbo Scientz, China) with ice-cold physiological saline. The clear supernatant liquid was obtained through centrifugation at 5000 rpm for 5 min at 4 °C and used for T-SOD, GSH, and MDA assays (Nanjing Jiancheng, Nanjing, China). The assays were conducted using the kits according to the protocols of the manufacturer. Total protein concentration in the supernatant was detected according to Coomassie Brilliant Blue method.

### Histopathological studies

Hematoxylin–eosin (H&E) staining was performed first. The liver tissue was fixed with 10% formalin at normal temperature, and was subsequently embedded in paraffin after 1 day. The tissues were cut into 5 μm-thick slices and then stained with H&E. Pathological section was examined in the blinding way under a light microscope.

Oil Red O staining was performed to observe the lipid droplet morphology as described in a previous study [38]. Quantitative analysis of the hepatic lipid content was conducted using Image-Pro Plus (Version 6.0).

### Statistical analysis

The results were reported as mean ± SEM. The data were analyzed using IBM SPSS Statistics 20. Statistically significant data were further analyzed and compared using Duncan’s multiple range test. Significant difference was considered at *p* < 0.05.

## Results

### General observation

When treated with 14 ml/kg alcoholic liquor, the rats in every group (except the normal control) behaved excitedly, such as unsteady walking and accelerated breathing. Food utilization simultaneously began to decrease. Except for the normal group, the other groups did not exhibit mortality until the 15th day after alcohol administration. Average weight dramatically declined in the groups treated with alcohol (Table 1). The result shows that Red Star Erguotou Liquor significantly reduced the BW of rats. No significant differences were found between the alcoholic liquor group and other treated alcoholic liquor groups. As shown in Table 1, liver and kidney weight to BW ratios were similar, and no significant change was observed among the different groups.

**Table 1:** Effect of YGMM on weight and organ index in control and alcohol-treated rats

Group	Dose	BW (g)		Liver index	Kidney index
		Initial	Final	(g/100 g BW^− 1^)	
Control		241.25 ± 6.50	419.78 ± 16.43	3.47 ± 0.07	0.56 ± 0.04
Alcohol		237.26 ± 4.59	346.73 ± 21.65^**^	3.32 ± 0.24	0.53 ± 0.01
Silymarin	100 mg/kg BW	237.16 ± 3.56	315.40 ± 24.93^***^	3.19 ± 0.12	0.55 ± 0.03
YGMM (low dose)	1.0 ml/kg BW	238.16 ± 3.46	310.88 ± 11.33^***^	3.71 ± 0.11	0.57 ± 0.03
YGMM (medium dose)	2.5 ml/kg BW	239.54 ± 4.24	335.73 ± 15.89^**^	3.50 ± 0.15	0.60 ± 0.04
YGMM (high dose)	5.0 ml/kg BW	234.26 ± 4.12	331.80 ± 16.51^**^	3.19 ± 0.08	0.61 ± 0.02

Data are presented as mean ± SEM in each group. Statistical significance is indicated by asterisks. **p < 0.01, ***p < 0.001 compared with normal control group. No significant differences were found between the alcohol group and other alcohol-treated groups

### Effect of YGMM on ALT and AST levels

As shown in Table 2, serum levels of AST and ALT in the alcohol group were significantly elevated after treatment with alcohol (*p* < 0.05), indicating the establishment of an acute alcohol-induced liver injury model in rats. YGMM exhibited a curative effect on the acute alcohol-induced liver injury by dose-dependently reducing the AST and ALT levels in rats. Finally, the AST and ALT levels of YGMM group (dose of 5 ml/kg) decreased by 40.86 and 24.71%, respectively, as compared with those in the alcohol group.

**Table 2:** Effect of YGMM on ALT, AST, TG and TB levels in control and alcohol-treated rats

Group	ALT (U/l)	AST (U/l)	TG (mg/dl)	TB (mg/dl)	AST/ALT
Control	8.49 ± 0.33	21.70 ± 1.10	3.75 ± 0.56	0.28 ± 0.02	2.57 ± 0.15
Alcohol	11.37 ± 0.37^##^	31.28 ± 1.95^###^	10.50 ± 2.14^##^	0.47 ± 0.04^###^	2.77 ± 0.23
Silymarin	8.09 ± 0.47^**^	24.16 ± 3.56^*^	2.67 ± 0.61^***^	0.32 ± 0.03^**^	3.03 ± 0.55
YGMM (low dose)	9.35 ± 1.12	22.24 ± 2.23^**^	3.08 ± 1.09^**^	0.33 ± 0.03^**^	2.41 ± 0.13
YGMM (medium dose)	8.34 ± 1.44^*^	19.79 ± 0.58^***^	4.77 ± 1.42^*^	0.26 ± 0.06^***^	2.52 ± 0.43
YGMM (high dose)	8.56 ± 0.41^*^	18.50 ± 1.88^***^	4.89 ± 1.80^*^	0.26 ± 0.01^***^	2.15 ± 0.14

Data are presented as mean ± SEM in each group. Statistically significant differences are indicated by asterisks and pound sign (^*^p < 0.05, ^**^p < 0.01, ^***^p < 0.001 compared with the model group; ^##^*p* < 0.01, ^###^*p* < 0.001 compared with the control group)

### Effect of YGMM on TB and TG levels

TB and TG contents in serum are presented in Table 2. TB and TG levels in the alcohol group were significantly increased. The administration of YGMM remarkably prevented the elevation of TB level in a dose-dependent manner. However, TG level did not show the same trend. No significant differences in TG level were found among YGMM groups, indicating that the effect of YGMM on TG level was not related to the dose within a certain concentration range.

### Effect of YGMM on T-SOD, GSH, and MDA levels

The T-SOD, GSH, and MDA levels in the liver and kidney are shown in Table 3. Compared with that in the control group, the GSH level in the alcohol group was significantly decreased (*p* < 0.05). However, T-SOD and MDA levels showed no significant change. The treatment of YGMM slightly recovered the decreased T-SOD and GSH levels. This obscure effect resulted from the mild oxidative damage in this study.

**Table 3:** Effect of YGMM on T-SOD, GSH, and MDA levels in alcoholic liver injury of rats

Group	Liver			Kidney		
	T-SOD (U/mg protein)	GSH (mg/g protein)	MDA (nmol/mg protein)	T-SOD (U/mg protein)	GSH (mg/g protein)	MDA (nmol/mg protein)
Control	214.67 ± 22.34	3.32 ± 0.37	1.53 ± 0.20	127.90 ± 15.81	3.95 ± 0.19	0.88 ± 0.04
Alcohol	180.71 ± 5.71	2.43 ± 0.24^#^	1.57 ± 0.11	99.43 ± 4.17	3.39 ± 0.11^#^	0.77 ± 0.04
Silymarin	199.52 ± 6.48	2.91 ± 0.11	1.57 ± 0.16	86.15 ± 3.30	3.03 ± 0.14	1.22 ± 0.07^***^
YGMM (low dose)	220.10 ± 18.50	3.18 ± 0.23^*^	1.61 ± 0.13	100.47 ± 14.39	3.72 ± 0.16	0.86 ± 0.06
YGMM (medium dose)	209.49 ± 8.93	2.86 ± 0.22	1.70 ± 0.24	85.07 ± 3.31	3.05 ± 0.19	0.73 ± 0.08
YGMM (high dose)	210.86 ± 19.61	2.96 ± 0.03	1.83 ± 0.13	96.94 ± 4.12	3.42 ± 0.15	0.47 ± 0.10^**^

Data are presented as mean ± SEM in each group. ^#^*p* < 0.05 compared with the normal control group; ^*^p < 0.05, ^**^p < 0.01, ^***^p < 0.001 compared with the alcohol group

### Histopathological studies

**Fig. 2:**
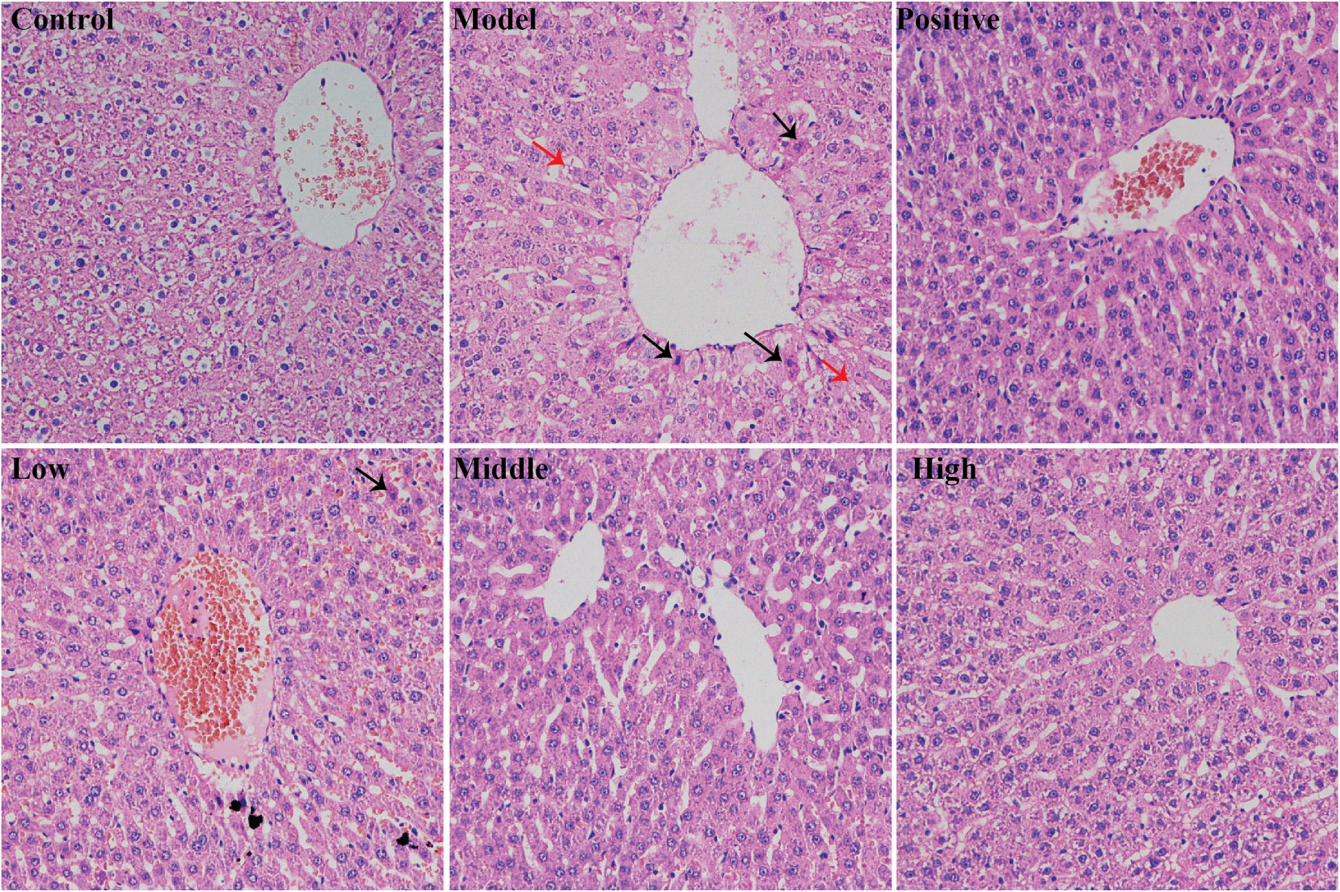
Light microscopic analysis of rat liver sections of normal rats and alcohol treatment with or without YGMM administration (H&E staining, 200×). Control group was given equal volumes of physiological saline, model group was treated with alcohol, positive group was treated with alcohol and Silymarin (100 mg/kg), low group was treated with alcohol and YGMM (1 ml/kg), middle group was treated with alcohol and YGMM (2.5 ml/kg), high group was treated with alcohol and YGMM (5 ml/kg). Black arrow showed inflammatory cells, and red arrow showed the swelling of liver cells

The livers of the normal control group exhibited resilience in a bright color after being separated from the rat body; however, those in the alcohol group were dim and swelling. The histological features are shown in Fig. 2. In the normal control group, the liver lobular architecture was clear, the central veins were intact, the hepatic cell cords were arranged neatly, and the cells were uniform in size. No pathological changes were detected in the normal control. However, the alcohol group showed some liver pathological changes, which are characterized by the cellular edema, inflammatory cell infiltrates. Histopathological changes caused by alcohol were improved by YGMM. The best therapeutic effect was achieved at a dose of 5 ml/kg, which was similar to the silymarin group and was remarkable distinction with the alcohol group.

**Fig. 3:**
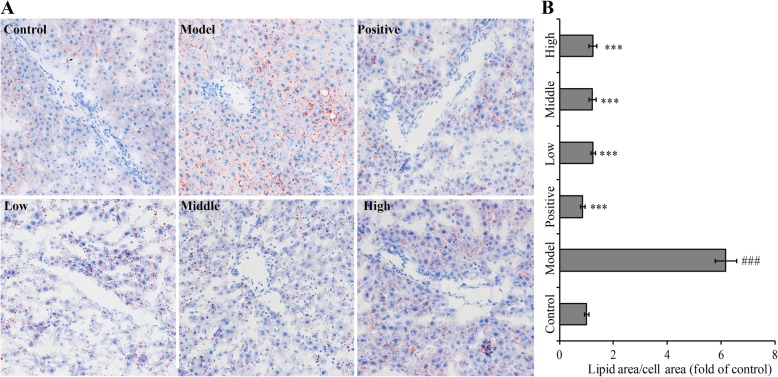
Oil Red O staining for qualitatively and quantitatively visualizing hepatic lipid accumulation under light microscope (200×). Control group was given equal volumes of physiological saline, model group was treated with alcohol, positive group was treated with alcohol and Silymarin (100 mg/kg), low group was treated with alcohol and YGMM (1 ml/kg), middle group was treated with alcohol and YGMM (2.5 ml/kg), high group was treated with alcohol and YGMM (5 ml/kg). ^###^*p* < 0.001 compared with the control group, ^***^p < 0.001 compared with the model group

In the liver tissues and cells, Oil Red O can dissolve in lipid combined with triglyceride and become orange lipid droplets. After staining with Oil Red O, abundant orange lipid droplets were found around the central veins in the alcohol group (Fig. 3 a). These lipid droplets were sharply reduced in the silymarin and YGMM groups (Fig. 3b), indicating that YGMM had a positive therapeutic effect for alcoholic fatty liver.

## Discussion

Many effective and accurate animal models have been developed to establish a liver pathology that is completely analogous to the clinical situation for ALD. Rodents and oral administration are the most suitable option for the animal model of ALD [39]. Wang et al. gave rats 50% alcohol at a dose of 12 ml/kg for 8 days [40], and another different method was that the mice orally received 2.4 g/kg of ethanol for 15 days [41]. Other researchers gave rats 6 g/kg of alcohol for 4 weeks [42]. In the preliminary assay, the serum levels of ALT, AST, and TG were all significantly increased after 4 weeks of alcohol treatment in rats. As a consequence, the experiment was terminated, and blood and liver and kidney tissues were collected on the 28th day of alcohol administration in our formal tests. Given the strong aversion of rats to alcohol, the consumption of animal feed was reduced after alcohol administration. In the present study, the BW of rats significantly decreased after alcohol treatment, which was not restored by YGMM (Table 1).

ALT and AST are important metabolic enzymes in liver cells and are usually at a low level in the plasma. When the structural integrity of hepatic cells and even organelles such as mitochondria were damaged from xenobiotics, soluble enzymes such as ALT and AST compartmented will be released into the blood [43]. Therefore, the serum transaminases (ALT and AST) usually are regarded as the optimum markers to diagnose liver injury [44]. In our study, significant increases of ALT and AST levels were obtained after administration with alcohol (Table 2), which indicated that alcohol treatment could damage the plasma and organelle membranes. YGMM pretreatment attenuated ALT and AST elevation in a dose-dependent manner (Table 2). The change complied with a universal viewpoint that the ALT and AST content recovered to a general level accompanied by liver parenchyma rehabilitation and hepatic cell regeneration [45].

Pyridoxal 5′-phosphate is an important ALT and AST coenzyme. Pyridoxal 5′-phosphate deficiency is common in ALD [46]. Its depletion causes a reduction of liver ALT at a certain degree than AST [47]. As a result, AST/ALT value is increased, which is regarded as typical in patients with alcoholic hepatitis [48]. This increased value in alcohol-related hepatic disease was found in 1967 [49]. The same changing trend could be found in people suffering from alcoholic hepatitis and acute liver injury in other studies [50, 51]. In the present study, the ratio of AST/ALT was increased in the alcohol group compared with normal control, which indicated an advanced ALD, although no significant change was observed (Table 2). A similar result was also reported by Lin et al. [42]. YGMM treatment inhibited the increase in AST/ALT ratio (Table 2), which may alleviate alcoholic hepatic injury.

Bilirubin is a final product of heme degradation by heme oxygenase. Biliverdin, which is converted by heme oxygenase, is the intermediate degradation product of bilirubin [52, 53]. Bilirubin is a potent antioxidant that can effectively remove ROS and nitric oxide [54, 55]. Accumulating evidence has shown that serum total bilirubin concentration is independently and inversely associated with the progression of diabetic nephropathy, coronary atherosclerosis, and type 2 diabetes with retinopathy [56–58]. In contrast to the aforementioned disorders, high serum total bilirubin concentration was observed in ALD and non-ALD patients [59, 60]. A possible mechanism for the increase in alcohol consumption is that alcohol competitively inhibits bilirubin conjugation [60]. UDP-glucuronosyltransferase (UGT1A1), a metabolizing enzyme preventing bilirubin accumulation to toxic levels [61], is involved in the pathway for glucuronidation of alcohol and bilirubin. Hence, the presence of alcohol will affect bilirubin conjugation by a competitive relationship. Although an elevation of UGT1A1 mRNA and microsomal UGT1A1 protein was observed in the liver of ethanol consumer animals [62], the compensation is not effective in the condition with high concentrations of alcohol substrate. In the current study, the alcohol group had a significant increase (167.86%) of serum total bilirubin level compared with the normal control group. However, YGMM treatment obviously recovered the serum total bilirubin content (Table 2). This therapeutic effect of YGMM may be due to stimulating the pathway for glucuronidation of bilirubin.

Studies show that fat accumulation is universal in hepatic cells treated with alcohol [63, 64]. Fat accumulation in ALD is a complicated process. The exact mechanisms underlying steatohepatitis, including fatty acid synthesis and oxidation, are primarily regulated by SREBP-1 and PPAR-α [65]. Alcohol inhibits PPAR-α transcriptional activating and DNA combining capacity, which is then influenced by dissociative fatty acid transportation and oxidization. Alcohol exposure also elevates SREBP-1 content and promotes the synthesis of fatty acids [65]. In the present study, the serum level of TG contents in the alcohol group was increased by 280% compared with that in the normal control group; however, YGMM treatment significantly reduced the serum TG level (Table 2). Oil Red O staining further confirmed the increase in lipid level (Fig. 3b). YGMM treatment (doses of 2.5 and 5 ml/kg) recovered the pathologic lesions similar to silymarin. The increase in serum TG level and lipid change in hepatocytes indicated that alcoholic fatty liver was already established in the animal model. In this study, YGMM treatment could cease fat accumulation caused by alcohol exposure. This therapeutic effect of YGMM may be facilitated through the above molecular pathways. Another possible pathway is the altered NADH/NAD^+^ redox potential, which has long been regarded as the way that alcohol causes fatty liver [65]. In this pathway, alcohol-induced fatty liver could be prevented by antioxidants [66]. Therefore, the effect of YGMM on serum TG level may explain clearly with the antioxidants contained in YGMM. Substantial alcohol may become the preferential fuel in hepatic cells and turn it into important energy source instead of fat, which supports fatty acid accumulation [67]. Hyperlipidemia prevails in a minimal liver damage at the beginning of alcohol consumption but has an opposite trend in a severe liver injury.

Oxidative stress, which is involved in alcohol abuse, can damage antioxidant defenses and produce ROS at the same time [68]. T-SOD, GSH, and MDA contents were measured in the liver and kidney tissues of each group to detect the oxidative damage induced by alcohol in the current study. Alcohol treatment exhibited 1.37-fold less GSH in liver tissues and 1.17-fold less GSH in kidney tissues compared with the control group. By contrast, the value of T-SOD and MDA showed no significant change. These data showed that mild oxidative damage was present in these two organs in the current assay. Thus, the hepatoprotective activity of YGMM for oxidative injury can be disregarded. In a previous study, a generous alcohol dose did not induce oxidative stress in livers of male SD rats [69]. Alcohol inhibited GSH synthesis and increased the consumption of GSH due to oxidation [70]. As a result, hepatic and nephritic GSH levels were decreased significantly in the current study. GSH is a key intracellular antioxidant [67], and increased consumption of GSH helps to keep a stabilized intracellular environment against pro-oxidants and antioxidants. Therefore, mild oxidative damage was obtained in this study. Alcohol-induced oxidative damage is considered to be a crucial player in the mechanisms by which alcohol produces liver injury. Cytochrome P450 2E1 is a critical enzyme in this process, which generates O_2_^•−^ and H_2_O_2_ [70]. O_2_^•−^ can be transformed to H_2_O_2_ by T-SOD, and then catalase transforms H_2_O_2_ to H_2_O. Another widely accepted idea, where MDA is closely related with cytochrome content, can be taken into consideration [71]. In this case, when oxidative damage was mild due to the increased consumption of GSH, the content of T-SOD and MDA ranged in the same level after alcohol treatment. No significant change in SOD level after alcohol administration (5 g/kg) to rats was observed by Develi et al. [72].

Yigan mingmu in Chinese means “benefits the liver and brightens the eyes.” To explore the hepatoprotective effect of YGMM, we established an acute alcohol-induced liver injury model. Our data revealed that YGMM treatment decreased significantly the high levels of ALT, AST, TB, and TG caused by alcohol exposure. Even so, the current work was the preliminary experimental study of YGMM under the ideal conditions, in which had the appropriate alcohol dose to induce the liver injury and proper volume of YGMM to prevent the progress of ALD. A better understand of YGMM to decrease fat accumulation needs to consider the human alcohol intake and dosage range of YGMM in daily life, and clinical experiments with more sample size. Meanwhile, further studies on the chemical composition analysis and molecular mechanism of hepatoprotective activity are necessary to understand the vital role of YGMM against acute alcohol-induced liver injury.

## Conclusion

YGMM exhibits an attractive protective effect against acute liver injury induced by alcohol in rats. The decreased ALT, AST, and TG levels indicated that YGMM could restore the damage in hepatic cell and cease fat accumulation. These properties are considered as the primary mechanisms of YGMM to prevent the progress of ALD. This study presented persuasive results to support the hepatoprotective activity of YGMM.

## Data Availability

All data generated or analysed during the current study are included in this article.

## Abbreviations

ALD: Alcoholic liver disease
ALT: Alanine transferase
AST: Aspartate transaminase
GSH: Glutathione
MDA: Malondialdehyde
PPAR-α: peroxisome proliferator-activated receptor-α
SREBP-1: sterol regulatory element binding protein 1
TB: Total bilirubin
TCM: Traditional Chinese Medicine
TG: Triglyceride
T-SOD: Total superoxide dismutase
